# Secular trends in overweight and obesity among urban children and adolescents, 2003–2012: A serial cross-sectional study in Guangzhou, China

**DOI:** 10.1038/s41598-017-12094-z

**Published:** 2017-09-21

**Authors:** Yinan Zong, Runsheng Xie, Nali Deng, Li Liu, Weiqing Tan, Yanhui Gao, Jiewen Yang, Yi Yang

**Affiliations:** 1 Department of Epidemiology and Biostatistics, School of Public Health, Guangdong Pharmaceutical University, Guangzhou, 510310 China; 2 Guangzhou Health Care Promotion Center for Primary and Middle Schools, Guangzhou, 510180 China

## Abstract

Childhood and adolescent overweight and obesity are increasing in China, but limited information is available on its secular trends in Guangzhou. In this cross-sectional study, ten-wave successive data were obtained from the physical fitness surveillance for students in Guangzhou from 2003 to 2012. A total of 2,619,154 urban students aged 7–18 years were included. The age-standardized prevalence of overweight and obesity increased significantly over the period: overweight rose from 10.15% to 14.07% in boys and 6.39% to 8.11% in girls, while obesity increased from 5.65% to 8.31% for boys and 3.43% to 4.12% for girls, respectively (*P* < 0.05). The increasing trend was significant across almost all age-sex-specific groups (*P* < 0.05), especially in the last five years. The prevalence of overweight and obesity grew continuously in both sexes, but the pace of change for boys were faster than that for girls. The highest prevalence of overweight was found among 10- to 12-year-old boys, that of obesity among 7- to 9-year-old boys and girls. In conclusion, overweight and obesity have increased significantly among urban children and adolescents in Guangzhou during 2003–2012. Further analysis of influencing factors and comprehensive interventions are urgently needed to combat the obesity epidemic among urban children and adolescents in Guangzhou.

## Introduction

The childhood and adolescent obesity epidemic is a global public health threat. Over the past 30 years, the prevalence of overweight and obesity in children and adolescents worldwide has shown an increase of 47.1%^1^. From 1980 to 2013, the combined prevalence of overweight and obesity in developed countries increased from 16.9% to 23.8% for boys and from 16.2% to 22.6% for girls; while in developing countries, the prevalence increased from 8.1% to 12.9% for boys and from 8.4% to 13.4% for girls^1^. Childhood and adolescent obesity significantly increase the risk of type 2 diabetes, hypertension, cardiovascular disease and liver disease^2–5^, which is likely to continue into adulthood^6,7^, leading to long-term health damage and increased morbidity and mortality in adults^8,9^. Considering the rapidly increasing prevalence and health risk, there is a need to monitor childhood and adolescent obesity continuously through population surveillance.

With its rapid economic development and dramatic transitions in lifestyle, China has undergone a significant increase in childhood and adolescent obesity^10–12^. According to the Chinese National Survey on Students’ Constitution and Health (CNSSCH), between 1985 and 2010, the combined prevalence of overweight and obesity in children aged 7 to 18 years increased from 1.0% to 20.0% in boys and from 1.6% to 11.5% in girls, which was equivalent to approximately 30.4 million overweight or obese children and adolescents of China in 2010^13^. Meanwhile, the epidemic of childhood and adolescent obesity varies in different regions of China. Based on the data from the China Health and Nutrition Survey (CHNS) in 2011 and the International Obesity Task Force (IOTF), Gordon-Larsen *et al*. reported that the overweight prevalence of children aged 2–18 years was 10.5% in Beijing, whereas the prevalence levels were 5.2% and 6.2% in Shanghai and Chongqing, respectively^14^. These regional differences may be due to the characteristics of the population’s genetic background, social economic structure or dietary and lifestyle patterns in local regions.

Guangzhou is a leading commercial region with a unique living culture in southern China. Whether its prevalence and trends of childhood and adolescent overweight and obesity are different from other regions is worth investigating. Several studies have provided the estimates of the prevalence and trends of overweight and obesity among children and adolescents in Guangzhou^15–18^, but yielded inconsistent results. Recent studies have reported the increasing trends of childhood and adolescent overweight and obesity^16–18^. However, Liu *et al*. have found similar change in the prevalence of obesity, but not overweight^15^. Ma *et al*. have reported that the prevalence of obesity for adolescents aged 12–18 years started to decrease during 2009–2011 in Guangzhou^17^, but other studies have not shown the same results^15,16,18^. Therefore, we conducted a serial cross-sectional analysis based on ten-wave physical fitness surveillance data of primary and secondary school students in Guangzhou during 2003–2012. The aims of this study were to describe the secular trends of overweight and obesity across different genders and age groups of Guangzhou urban children and adolescents, as well as to compare the growth rate of overweight and obesity in different periods.

## Materials and Methods

### Data sources and study population

Data were obtained from the physical fitness surveillance for students in Guangzhou, which is a sequential cross-sectional investigation conducted annually by the Guangzhou Health Care Promotion Center for Primary and Middle Schools since 2003. After excluding students with missing data on sex, age, height, or weight (n = 1,278), we enrolled a total of 2,619,154 urban students aged 7–18 years during the period of 2003–2012. The average number of study population each year were equal to 17% of Guangzhou urban children and adolescents aged 5 to 19 years (261,915/1,580,818) according to the 2010 Census^19^. The sex composition of the study population resembled that of all children and adolescents in Guangzhou (53.7% boys, 46.3% girls)^19^. The sex and age distributions of the study population during 2003–2012 are shown in Table 1.

**Table 1:** Distribution of the study population by sex and age groups, 2003–2012.

Subgroup	Year									
	2003	2004	2005	2006	2007	2008	2009	2010	2011	2012
**All (n)**	225,199	262,704	270,992	290,043	273,865	274,726	263,642	267,823	264,683	225,477
**Boys (n)**										
7–9 y	14,884	17,205	16,869	17,065	17,825	18,913	18,057	17,670	18,298	16,244
10–12 y	17,275	19,913	20,767	22,785	24,130	25,584	24,684	26,518	26,115	23,805
13–15 y	48,488	54,192	52,943	59,250	53,608	54,312	53,345	55,417	55,325	43,005
16–18 y	32,190	40,982	45,814	47,225	43,553	41,186	37,962	37,215	35,945	31,740
Total	112,837	132,292	136,393	146,325	139,116	139,995	134,048	136,820	135,683	114,794
**Girls (n)**										
7–9 y	12,989	14,488	14,308	14,302	15,114	16,051	15,197	14,803	15,312	13,627
10–12 y	15,723	17,800	18,807	20,477	21,309	22,599	21,923	23,116	23,049	21,267
13–15 y	46,138	51,448	50,098	55,903	50,114	50,517	50,077	52,032	50,982	40,513
16–18 y	37,512	46,676	51,386	53,036	48,212	45,564	42,397	41,052	39,657	35,276
Total	112,362	130,412	134,599	143,718	134,749	134,731	129,594	131,003	129,000	110,683

Ethics approval for this study was granted by the Institutional Ethics Committee of Guangdong Pharmaceutical University, Guangzhou, China (Medical Ethics Review [2016] No. 17). Written informed consent was obtained from all surveillance subjects or their parents. This study was performed in line with the principles of the Declaration of Helsinki, and in accordance with all relevant guidelines and regulations for medical research.

### Measurements

Measurements of the height and weight were performed by well-trained health professionals in each study year. Before measurements, participants were asked to wear underclothes only and to stand upright without shoes. Height was measured to the nearest 0.1 centimeter (cm) with a metal column height measuring stand, and weight was measured to the nearest 0.1 kilogram (kg) with a calibrated beam scale. The body mass index (BMI) was calculated as the body weight in kilograms divided by the square of height (kg/m^2^); then, overweight and obesity were defined by the BMI Reference for Screening Overweight and Obesity in Chinese School-aged Children (WGOC-BMI criteria), which was established by the Working Group of Obesity in China (WGOC)^20,21^. In the WGOC-BMI criteria, children with BMI values greater than or equal to the 85th and 95th percentile of the age-sex-specific BMI are defined as overweight and obese, respectively. For both boys and girls aged 18 years, BMI of 24 and 28 kg/m^2^ are the cutoffs for overweight and obesity, respectively, which meets the Chinese cut-points for adult^20^.

### Statistical analysis

The mean BMI and prevalence of overweight and obesity in each study year were calculated by sex and age groups (7–9 y, 10–12 y, 13–15 y, 16–18 y). The distribution change in the BMI between 2003 and 2012 was evaluated by the Kernel-density curve, which is a nonparametric smoothed graph. The age- standardized prevalence was calculated by the direct method using the national data of the China Census 2010 as a standard population. General linear model was used to test secular trends in mean BMI, and the prevalence of overweight or obesity for children and adolescents during 2003–2012. To further examine the magnitude of BMI changes and the prevalence of overweight and obesity, we also calculated the annual growth rate (%) during 2003–2007, 2008–2012, and 2003–2012 using the following equation (1).

1 $${\rm{Annual}}\,{\rm{growth}}\,{\rm{rate}}=(\sqrt[{n}_{2}-{n}_{1}]{{a}_{{n}_{2}}/{a}_{{n}_{1}}}-1)\ast 100 \% $$

*n* is the study year, and *a* is the prevalence of overweight or obesity in *n* year. Data analyses were performed using SAS Version 9.4 (SAS Institute Inc., Cary, NC, USA). A *P* value < 0.05 was considered statistically significant.

### Data availability statement

The datasets generated during and/or analysed during the current study are available from the corresponding author on reasonable request.

## Results

### Secular changes in BMI

**Figure 1:**
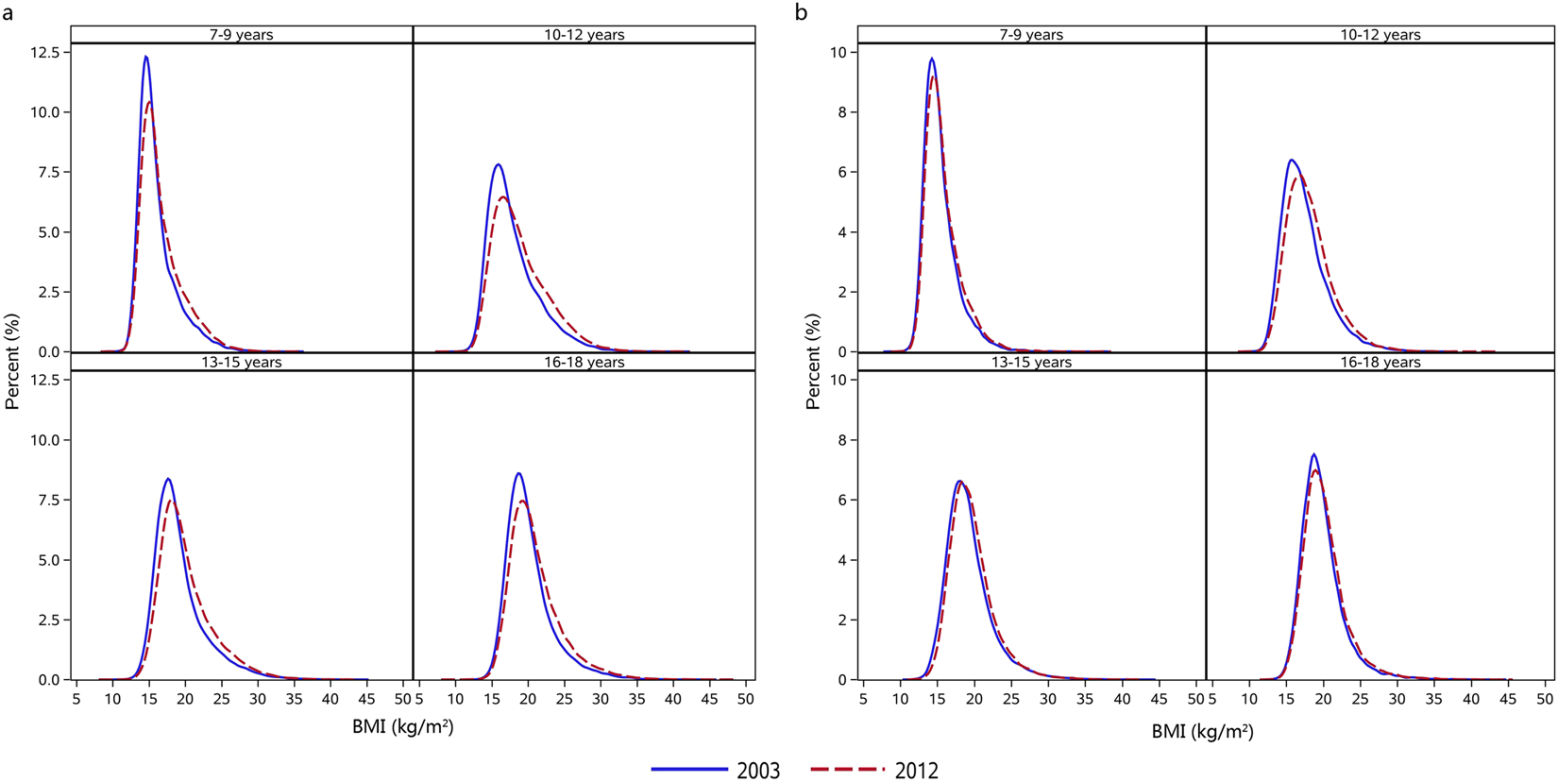
Distribution change of body mass index in urban boys (a) and girls (b) aged 7–18 years in Guangzhou, 2003–2012.

From 2003 to 2012, the mean BMI of boys increased from 18.86 kg/m^2^ to 19.57 kg/m^2^, while it increased from 18.71 kg/m^2^ to 19.01 kg/m^2^ for girls (Table 2). Similar increasing trends were observed across all age groups in both sexes (*P* < 0.05), with an average annual growth ranging from 0.20% to 0.59% (Table 2). Compared with girls, the mean BMI of boys increased slightly faster in each age group. As shown in Fig. 1, the BMI distribution curves for boys shifted to the right between 2003 and 2012, and their upper tails were somewhat elevated. In girls, the BMI distribution had no obvious changes from 2003 to 2012.

**Table 2:** Mean body mass index (kg/m^2^) in urban children and adolescents aged 7–18 years in Guangzhou, China, 2003–2012.

Subgroup	Year										β	SE	Annual growth rate (%)		
	2003	2004	2005	2006	2007	2008	2009	2010	2011	2012			2003–2007	2008–2012	2003–2012
Boys															
7–9 y	16.06	16.04	16.06	16.16	16.22	16.40	16.57	16.48	16.47	16.65	0.072^a^	0.009	0.25	0.38	0.40
10–12 y	17.90	17.90	17.91	18.07	18.13	18.31	18.50	18.53	18.61	18.87	0.111^a^	0.009	0.32	0.76	0.59
13–15 y	19.21	19.28	19.25	19.26	19.26	19.51	19.67	19.73	19.82	20.04	0.092^a^	0.012	0.07	0.67	0.47
16–18 y	20.15	20.18	20.17	20.20	20.25	20.43	20.63	20.70	20.72	20.95	0.092^a^	0.010	0.12	0.63	0.43
Total	18.86	18.93	18.96	19.02	18.98	19.14	19.31	19.34	19.37	19.57	0.072^a^	0.008	0.16	0.56	0.41
Girls															
7–9 y	15.41	15.34	15.22	15.35	15.44	15.56	15.63	15.58	15.51	15.72	0.041^a^	0.010	0.05	0.26	0.22
10–12 y	17.44	17.36	17.34	17.48	17.58	17.72	17.76	17.85	17.87	18.10	0.079^a^	0.009	0.20	0.53	0.41
13–15 y	19.22	19.16	18.97	19.18	19.14	19.36	19.31	19.43	19.48	19.62	0.053^a^	0.012	−0.10	0.33	0.23
16–18 y	19.78	19.73	19.54	19.64	19.74	19.81	19.84	19.94	19.95	20.13	0.045^a^	0.012	−0.05	0.40	0.20
Total	18.71	18.69	18.56	18.73	18.69	18.78	18.79	18.88	18.86	19.01	0.035^a^	0.008	−0.03	0.30	0.18

Beta coefficient (*β*) and standard error (SE) were calculated by general linear model.

^a^ *P* value < 0.05.

### Secular trends in overweight prevalence

**Figure 2:**
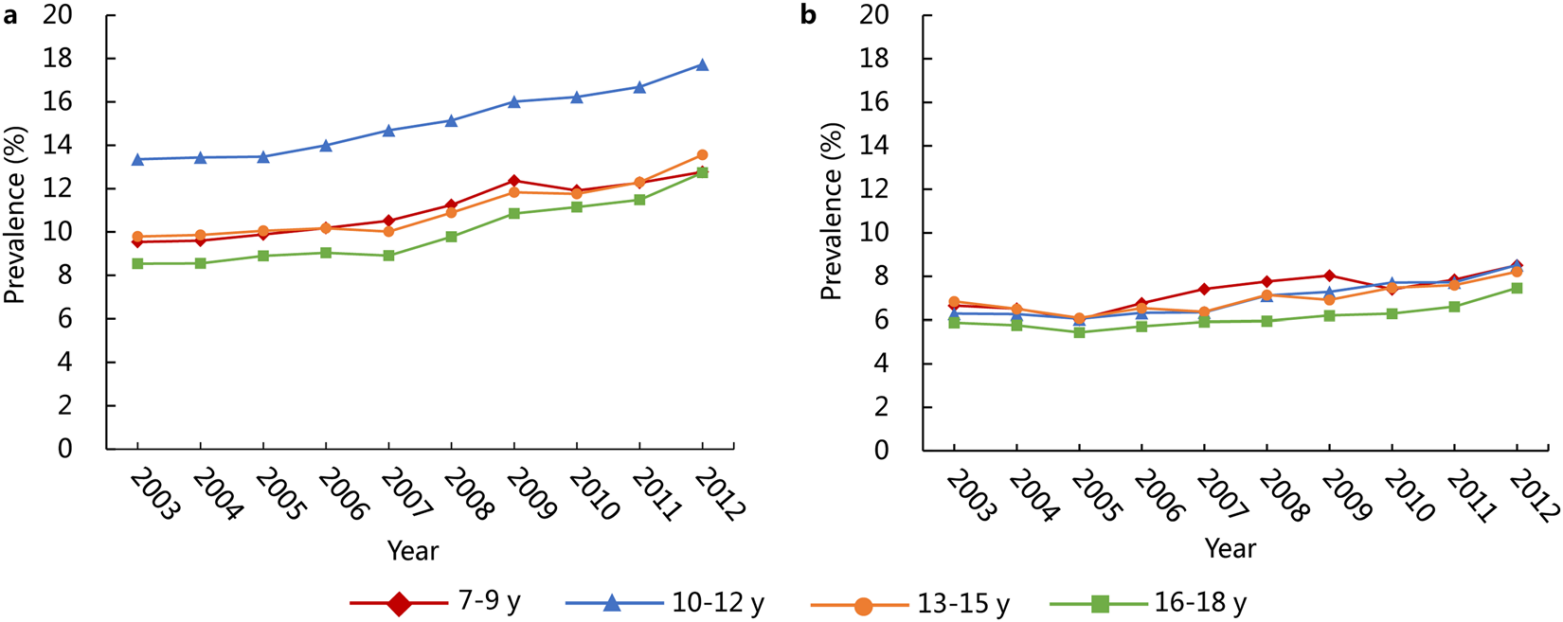
Secular trends in prevalence of overweight in urban boys (a) and girls (b) aged 7–18 years in Guangzhou, 2003–2012. Overweight was defined by the WGOC-BMI criteria.

During the 10-year period, the overweight prevalence increased significantly in children and adolescents in Guangzhou (*P* < 0.05; Table 3). For boys, the age-standardized overweight prevalence increased from 10.15% to 14.07%, and for girls, it increased from 6.39% to 8.11%, with average annual growth rates of 3.70% and 2.69% for boys and girls, respectively. Trends over time are presented in Fig. 2, and clear increases were seen across all age groups in both sexes. Obviously, for boys, the prevalence of overweight was much higher than that of the same age group in girls, and it showed more rapid growth in boys. Boys aged 10–12 years showed the highest overweight prevalence consistently, which reached 17.73% in 2012, whereas the prevalence values of other groups were less than 14%. However, when analyzing the average annual growth of different age groups for each sex, the fastest growth occurred in boys aged 16 to 18 years (4.54%) and girls aged 10 to 12 years (3.43%). The annual growth rates were obviously higher in the last five years (2008–2012) than in the first five years (2003–2007) for boys and for most age groups of girls (except for girls aged 7 to 9 years).

**Table 3:** The prevalence of overweight (%) in urban children and adolescents aged 7–18 years in Guangzhou, China, 2003–2012.

Subgroup	Year										β	SE	Annual growth rate (%)		
	2003	2004	2005	2006	2007	2008	2009	2010	2011	2012			2003–2007	2008–2012	2003–2012
Boys															
7–9 y	9.54	9.60	9.88	10.18	10.52	11.25	12.37	11.91	12.27	12.77	0.395^a^	0.036	2.47	3.22	3.29
10–12 y	13.35	13.44	13.48	14.00	14.69	15.14	16.01	16.23	16.70	17.73	0.500^a^	0.035	2.42	4.03	3.20
13–15 y	9.79	9.86	10.05	10.17	10.02	10.89	11.83	11.76	12.29	13.56	0.396^a^	0.053	0.58	5.64	3.69
16–18 y	8.54	8.55	8.90	9.04	8.91	9.78	10.85	11.15	11.48	12.73	0.459^a^	0.054	1.07	6.81	4.54
Total*	10.15	10.20	10.43	10.68	10.83	11.58	12.58	12.61	13.03	14.07	0.439^a^	0.041	1.64	5.00	3.70
Girls															
7–9 y	6.66	6.52	6.03	6.76	7.42	7.76	8.04	7.40	7.84	8.51	0.224^a^	0.045	2.74	2.33	2.76
10–12 y	6.29	6.26	6.05	6.32	6.35	7.12	7.29	7.71	7.73	8.52	0.257^a^	0.036	0.24	4.59	3.43
13–15 y	6.85	6.50	6.09	6.54	6.37	7.14	6.92	7.48	7.59	8.21	0.174^a^	0.044	−0.18	3.55	2.03
16–18 y	5.89	5.77	5.44	5.72	5.92	5.96	6.21	6.29	6.61	7.46	0.156^a^	0.037	0.13	5.77	2.66
Total*	6.39	6.22	5.87	6.28	6.45	6.90	7.02	7.15	7.37	8.11	0.198^a^	0.034	0.25	4.12	2.69

Overweight was defined by WGOC-BMI criteria.

*The total prevalence of overweight was age-standardized by the direct method to the 2010 China’s Census population using the 7–9 y, 10–12 y, 13–15 y, and 16–18 y age groups. Crude prevalence can be found as in Supplementary Table S1.

Beta coefficient (*β*) and standard error (SE) were calculated by general linear model.

^a^ *P* value < 0.05.

### Secular trends in obesity prevalence

**Figure 3:**
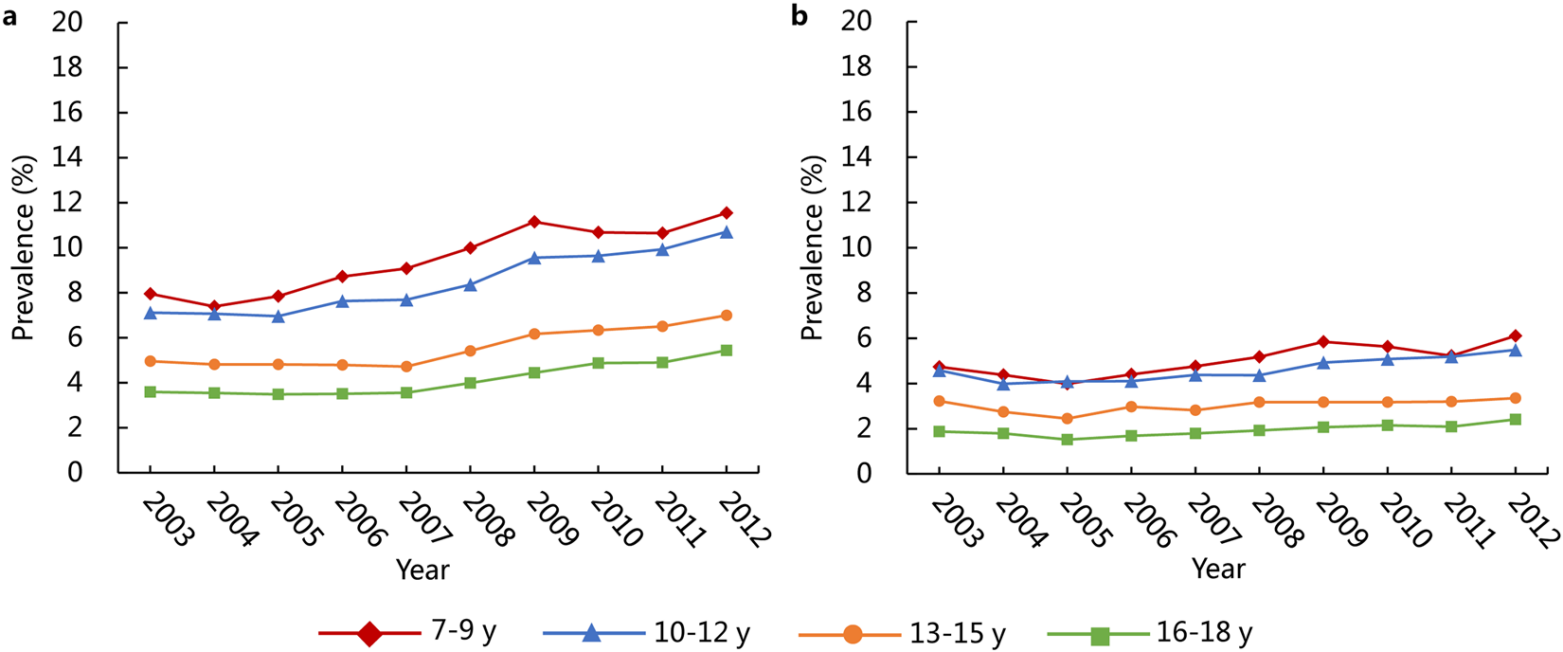
Secular trends in prevalence of obesity in urban boys (a) and girls (b) aged 7–18 years in Guangzhou, 2003–2012. Obesity was defined by the WGOC-BMI criteria.

As with the trend for overweight, the prevalence of obesity also increased considerably in Guangzhou over the 10-year period (*P* < 0.05; Table 4). The age-standardized prevalence of obesity was 8.31% for boys and 4.12% for girls in 2012, and it was 5.65% for boys and 3.43% for girls in 2003. Sex disparity in the trends and levels of obesity was also obvious, as shown in Fig. 3. Although the prevalence of obesity increased across almost all age groups in both sexes (except girls aged 13–15 years with *P* > 0.05), it increased faster in boys than in girls (annual growth rate: 4.37% vs. 2.08%). Moreover, the obesity prevalence for girls was approximately half of that for boys in each year. When comparing their annual growth rates, boys aged 16–18 years and girls aged 7–9 years experienced the fastest increase in obesity prevalence, whereas the lowest growth rates for boys and girls were present at 13–15 years. The more rapid increase in the obesity prevalence mainly occurred in the last five years for boys and girls compared with fist five years. In addition, the obesity prevalence declined with age in each study year.

**Table 4:** The prevalence of obesity (%) in urban children and adolescents aged 7–18 years in Guangzhou, China, 2003–2012.

Subgroup	Year										β	SE	Annual growth rate (%)		
	2003	2004	2005	2006	2007	2008	2009	2010	2011	2012			2003–2007	2008–2012	2003–2012
Boys															
7–9 y	7.95	7.39	7.84	8.71	9.08	9.98	11.15	10.68	10.64	11.54	0.470^a^	0.055	3.38	3.70	4.23
10–12 y	7.11	7.06	6.96	7.62	7.69	8.36	9.55	9.64	9.92	10.70	0.438^a^	0.044	1.98	6.36	4.65
13–15 y	4.96	4.81	4.81	4.79	4.72	5.41	6.17	6.33	6.50	6.99	0.258^a^	0.043	−1.23	6.62	3.89
16–18 y	3.59	3.54	3.48	3.51	3.56	3.98	4.44	4.87	4.90	5.43	0.220^a^	0.034	−0.21	8.08	4.70
Total*	5.65	5.47	5.53	5.86	5.96	6.60	7.45	7.55	7.65	8.31	0.332^a^	0.036	1.30	5.92	4.37
Girls															
7–9 y	4.73	4.38	3.98	4.40	4.76	5.18	5.84	5.63	5.22	6.10	0.189^a^	0.045	0.16	4.17	2.87
10–12 y	4.58	3.99	4.09	4.10	4.38	4.36	4.93	5.08	5.19	5.49	0.146^a^	0.033	−1.11	5.93	2.03
13–15 y	3.22	2.75	2.45	2.97	2.82	3.17	3.18	3.18	3.20	3.35	0.054	0.026	−3.26	1.39	0.44
16–18 y	1.87	1.79	1.52	1.68	1.79	1.92	2.07	2.15	2.09	2.41	0.069^a^	0.018	−1.09	5.85	2.86
Total*	3.43	3.08	2.86	3.13	3.26	3.48	3.79	3.81	3.73	4.12	0.107^a^	0.026	−1.22	4.36	2.08

Obesity was defined by WGOC-BMI criteria.

*The total prevalence of obesity was age-standardized by the direct method to the 2010 China’s Census population using the 7–9 y, 10–12 y, 13–15 y, and 16–18 y age groups. Crude prevalence can be found as in Supplementary Table S1.

Beta coefficient (*β*) and standard error (SE) were calculated by general linear model.

^a^ *P* value < 0.05.

## Discussion

In this study, our results indicated that the mean BMI and the prevalence of overweight and obesity increased significantly across almost all age-sex-specific groups of Guangzhou urban children and adolescents during 2003–2012, especially for boys. This increase was more rapid during the five-year period of 2008–2012. This finding is a warning that the overweight and obesity epidemic is accelerating among urban children and adolescents aged 7–18 years in Guangzhou. To the best of our knowledge, this study is the first investigation using successive population-based surveillance data of a large sample size to examine the secular trends in childhood and adolescent overweight and obesity in Guangzhou, and it provides useful information to decision-making in the prevention and control of overweight and obesity.

In recent decades, Chinese children and adolescents have experienced a positive secular trend in height, weight and BMI^22^. The increase in BMI mainly occurred in their upper percentile distribution^23,24^, which means that the proportion of overweight and obesity was increasing. Across urban areas in China, the prevalence of overweight increased from 10.7% to 14.6% for boys and from 6.3% to 8.6% for girls during 2000 to 2010; for obesity in the same period, the prevalence increased from 5.0% to 8.6% and from 2.8% to 4.1% for boys and girls, respectively^25^. Consistent with this nationwide observation, our results indicate the age-standardized prevalence of overweight increased from 10.2% to 14.1% and from 6.4% to 8.1% for boys and girls, and the prevalence of obesity was 8.3% for boys and 4.1% for girls in 2012 compared with 5.7% and 3.4% in 2003, respectively. Of note, some studies have reported that the growth rate of overweight and obesity has slowed down slightly in Chinese children and adolescents from 2005 to 2010 compared to before 2005^26,27^. A recent study from Guangzhou has found that the prevalence of obesity among adolescents aged 12–18 years decreased between 2009 and 2011^17^. However, there is no similar change in this study. Although the prevalence of urban children and adolescent overweight and obesity was relatively steady during 2009–2011, the significant increase was observed in their prevalence in 2012. Overall, the growth of overweight and obese children and adolescents have accelerated in Guangzhou urban areas during the last five years (2008–2012). The cause of this increasing trend may be the more obesogenic environment brought by rapid change of dietary and physical activity patterns^28,29^, but in order to have a clearer understanding, prospective studies are warranted.

Nevertheless, compared with northern regions of China, Guangzhou’s prevalence level was lower. In Shandong province, for example, the prevalence of obesity reached 15.8% for boys and 7.1% for girls in 2010^30^. In Beijing, the prevalence of obesity in 2010 reached 17.1% and 11.9% for boys and girls, respectively^27^. Ji *et al*. suggested that this demographic disparity was partially due to the complex interaction of geographic-climate factors between northern and southern China^31^. People living in the warm and wet environment of southern China are more likely to have different dietary habits from their counterparts living in northern China where the climate is much colder and drier. For example, most Guangzhou residents have a lighter diet, less fatty diet, and like drinking soup before meals^31^.

Many other countries, such as India^32^, Northern Israel^33^, Scotland^34^, and Korea^35^, have also witnessed an increasing trend in childhood overweight and obesity. In India, the prevalence of obesity in urban Asian adolescents aged 14–17 years increased significantly from 9.8% to 11.7% between 2006 and 2009^32^. In general, the combined prevalence of overweight and obesity among Guangzhou boys was close to the average level of developed countries (22.4% in Guangzhou vs. 23.8% in developed countries) but this prevalence among Guangzhou girls was still very low and even slightly lower than the average level of developing countries (12.2% in Guangzhou vs. 13.4% in developing countries) according to the Global Burden of Disease Study^1^. Notably, stabilizing or declining trends in childhood and adolescent obesity have been reported in some developed countries, including the US^36^, the UK^37^, and Australia^38^, in recent years. The US National Health and Nutrition Examination Surveys declared no significant change in obesity among children and adolescents aged 2–19 years^36^. The childhood and adolescent obesity epidemic in these developed countries appeared earlier and has received sufficient attention, which has inspired various programs and policies for preventing obesity, such as Get Healthy Philly in Pennsylvania, a community-wide effort to improve public nutrition and physical activity^39^, and the “Eat Well Play Hard” program of New York, which aims to provide positive messages about eating healthy foods and being physically active to pre-school children and their parents^40^. These positive achievements in developed countries may provide helpful references for addressing childhood and adolescent obesity in Guangzhou.

Although the prevalence of overweight and obesity in Guangzhou grew significantly across almost all age groups for boys and girls, we observed remarkable gender and age disparity. The prevalence level of overweight and obesity for boys was dramatically higher than those for girls. Similar gender disparity was a common phenomenon in China according to previous studies^12,14,41^, but these prevalence levels for Guangzhou girls were relatively more stable and have only recently begun to increase. In many other countries, such as the US^42^ and the UK^43^, there were no significant gender differences. On the contrary, in South Africa, overweight and obesity only increased in girls^44^. We also found that boys aged 10–12 years had the highest prevalence of overweight during this decade, and the prevalence of obesity decreased with age in boys and girls, while the obesity growth rate was more rapid in 16- to 18-year-old adolescents. These characteristics were similar to the findings in previous studies in China^26,41^, but they contrasted with data from the UK, where the prevalence of obesity increased with age^43^. The gender and age disparity may be related to socio-cultural differences, body image perceptions and genetic factors^41,45^, but further research is needed.

This study used ten-wave successive surveillance data from 2003 to 2012, which contained a substantial number of the study population in each study year. Moreover, to ensure the comparability between different years, the crude prevalence rates were standardized according to the national population data of China Census 2010. The above features were critical to the reliability of the conclusion. As a limitation, the data used in the study were acquired from cross-sectional surveillance, which could not follow up the weight change in the individual level and assessment of the cohort and time effect. However, it was sufficient to reveal the epidemic level and secular trends.

In conclusion, this serial cross-sectional study indicated significant increasing trends in the prevalence of overweight and obesity among Guangzhou urban children and adolescents during 2003–2012, especially in the last five years. Considering childhood and adolescent overweight and obesity have been a major public issue, further study should explore related factors in order to make comprehensive interventions for controlling overweight and obesity among urban children and adolescents in Guangzhou.

## Electronic supplementary material

Supplementary Table S1.
