# Supplementary material for: Secular trends in overweight and obesity among urban children and adolescents, 2003–2012: A serial cross-sectional study in Guangzhou, China
Source: Sci Rep. 2017 Sep 21;7:12042. doi: 10.1038/s41598-017-12094-z (PMC5608869; doi:10.1038/s41598-017-12094-z)
Supplement: Supplementary file 1 — Supplementary Table S1. [file 41598_2017_12094_MOESM1_ESM.pdf]

Secular trends in overweight and obesity among urban children and adolescents, 2003-2012: A serial cross-sectional study in Guangzhou, China.

Yinan Zong[1*], Runsheng Xie[1*], Nali Deng[2], Li Liu[1], Weiqing Tan[2], Yanhui Gao[1], Jiewen Yang[2] & Yi Yang[1]

[1]Department of Epidemiology and Biostatistics, School of Public Health, Guangdong Pharmaceutical University, Guangzhou 510310, China

[2]Guangzhou Health Care Promotion Center for Primary and Middle Schools, Guangzhou 510180, China.

*These authors contributed equally to this work.

Correspondence and requests for materials should be addressed to Y.Y. (email: yangyigz@163.com) or J.Y. (email: 360449586@qq.com)

Supplementary Table S1. The crude prevalence of overweight and obesity (%) in urban boys and girls aged 7-18 years in Guangzhou, 2003-2012

Subgroup	Year									
	2003	2004	2005	2006	2007	2008	2009	2010	2011	2012
**Overweight**										
Boys	9.94	9.96	10.16	10.41	10.55	11.39	12.39	12.48	12.92	14.08
Girls	6.43	6.21	5.83	6.23	6.32	6.81	6.88	7.14	7.34	8.07
**Obesity**										
Boys	5.29	5.09	5.07	5.28	5.43	6.15	6.98	7.14	7.29	7.97
Girls	3.13	2.76	2.49	2.80	2.92	3.19	3.43	3.47	3.45	3.80

Overweight and obesity were defined by WGOC-BMI criteria.
